# *Propionibacterium acnes* evades microbicidal phagocytosis by inhibiting the mitochondrial biogenesis of nucleus pulposus cells

**DOI:** 10.1111/febs.70247

**Published:** 2025-09-02

**Authors:** Lemeng Ren, Changwei Li, Juntao Sun, Yuehuan Zheng, Yucheng Jiao, Jiancheng Zheng, Fangke Zhang, Yazhou Lin, Wenjian Wu, Peng Cao

**Affiliations:** ^1^ Department of Orthopedics, Ruijin Hospital Shanghai Jiaotong University School of Medicine China; ^2^ Shanghai Key Laboratory for Prevention and Treatment of Bone and Joint Diseases with Integrated Chinese‐Western Medicine, Shanghai Institute of Traumatology and Orthopedics, Ruijin Hospital Shanghai Jiaotong University School of Medicine China; ^3^ Department of Gastroenterology, The Second Hospital, Cheeloo College of Medicine Shandong University Jinan China; ^4^ Department of Orthopaedics The First Affiliated Hospital of Wenzhou Medical University China

**Keywords:** bactericidal, intervertebral disc degeneration, mitochondrial biogenesis, mitochondrial reactive oxygen species, *Propionibacterium acnes*

## Abstract

Although an increasing number of investigators confirm the latent infection of *Propionibacterium acnes* in degenerated nucleus pulposus tissue, the molecular mechanism by which *P. acnes* evades being eliminated and establishes persistent colonization in the nucleus pulposus (NP) tissue remains unknown. In this study, we ascertained that despite the resistance by nucleus pulposus cells (NPCs) to the invasion of *P. acnes* through microbicidal phagocytosis, *P. acnes* is able to nevertheless promote its long‐term colonization by inhibiting the sustained bactericidal capability of NPCs. This allows *P. acnes* to reside in intervertebral discs for an extended period, ultimately inducing chronic infectious intervertebral disc degeneration (IVDD). Mechanistically, *P. acnes* impairs the mitochondrial biogenesis of NPCs through the AMPK/SIRT‐1/PGC‐1α signaling pathway. This results in impaired mitochondria that are unable to generate sufficient ATP and deliver mitochondrial reactive oxygen species (mROS) to carry out the bactericidal process effectively, thus hampering the sustained microbicidal function. These findings provide novel insights into how *P. acnes* evades being phagocytosed and killed by NPCs and may offer potential therapeutic targets for the treatment of infectious IVDD.

## Graphical abstract

*P. acnes* infection impairs the mitochondrial biogenesis of nucleus pulposus cells (NPCs) through the AMPK/SIRT‐1/PGC‐1α signaling pathway. This results in impaired mitochondria that are unable to generate sufficient ATP, and impaired delivery of mitochondrial reactive oxygen species to carry out the bactericidal process effectively. Consequently, this compromises sustained microbicidal function, ultimately inducing chronic infectious intervertebral disc degeneration.

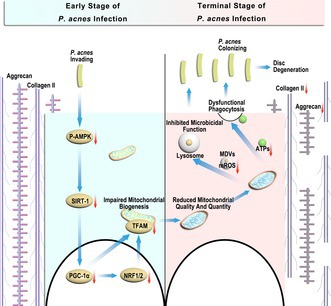

**Abbreviations**
18S rDNA: 18S ribosomal DNA
AMPK: AMP‐activated protein kinase
COL2: collagen II
COX1: cytochrome c oxidase subunit 1
COX2: cytochrome c oxidase subunit 2
DEGs: differentially expressed genes
IVDD: intervertebral disc degeneration
IVDs: intervertebral discs
MDVs: mitochondrial‐derived vesicles
MOI: multiplicity of infection
mROS: mitochondrial reactive oxygen species
mtDNA: mitochondrial DNA
nDNA: nuclear DNA
NP: nucleus pulposus
NPCs: nucleus pulposus cells
NRF1: nuclear respiratory factor 1
NRF2: nuclear factor erythroid 2‐related factor 2
OCR: oxygen‐consumption rate
*P. acnes*
: *Propionibacterium acnes*
PGC‐1α: peroxisome proliferator‐activated receptor gamma coactivator‐alpha
SIRT‐1: sirtuin 1
TFAM: transcription factor A, mitochondrial

## Introduction

Degenerative disc disease is the most common spinal disorder, often leading to a series of chronic clinical conditions such as sciatica and lower back pain, and significantly impacts the quality of life and work abilities of patients [1]. Therefore, discussions of its causes and treatments have stirred considerable interest in orthopedics.

The degeneration of intervertebral discs (IVDs) is influenced by multiple factors and processes, including excessive mechanical load, diseases of nutritional deficiency, trauma, and genetic predisposition [2]. As research progresses, bacterial infections—particularly chronic infections by low‐toxic anaerobic bacteria—are gaining attention with respect to IVDs [3]. In 2001, Stirling et al. [4] were the first to publish on the colonization of *P. acnes* in non‐purulent degenerated IVDs in *The Lancet*, speculating that its latent infection caused persistent low back pain and nerve root lesions due to lumbar disc herniation. This hypothesis has since garnered significant attention. In 2013, Albert et al.'s [5] randomized controlled study revealed that antimicrobial therapy effectively alleviated chronic low back pain, highlighting the relationship between low‐toxic bacterial infection and IVDD. While some scholars question whether low‐toxic bacteria truly carry latent infections within degenerated discs [6], an increasing number of studies from different perspectives have reported the presence of low‐toxic anaerobic bacteria in degenerated IVDs, with a positivity rate of 11.3% to 44%, mainly involving *P. acnes* and coagulase‐negative staphylococci [7, 8, 9, 10, 11]. Research findings from multiple independent teams have also confirmed that colonization of *P. acnes* can promote or cause the onset of IVDD. This colonization induces chronic inflammation of the nucleus pulposus cells [9, 12] and oxidative damage [13, 14], promotes degradation of the extracellular matrix [15], and induces apoptosis of NPCs [16].

Innate immunity serves as the body's initial defense against microbial infections, with specialized phagocytic cells such as neutrophils, monocytes, and macrophages quickly mobilized to eliminate invading bacteria such as *Staphylococcus aureus* [17]. Multiple studies have shown that NPCs exhibit phagocytic cell characteristics, expressing markers such as CCR7, CD163, and CD206 on their membranes [18]. *In vitro* studies entailing electron microscopy have shown that NPCs are capable of forming pseudopods similar to those in phagocytic cells [19]. Moreover, our previous research indicated that NPCs effectively engulfed bacteria such as *Staphylococcus aureus* and *P. acnes*, forming phagolysosomes that led to bacterial degradation over time [20]. However, it is important to emphasize that NPCs were unable to completely eradicate invading *P. acnes*, and this finding is consistent with clinical observations of *P. acnes* detected in NP tissue of over 20% of patients suffering from chronic disc degeneration and lower back pain. We therefore asked, what are the key molecular mechanisms that enable *P. acnes* to establish long‐term colonization in NP tissue, and could the phenomenon be linked to the suppression of microbicidal phagocytosis function by NPCs?

We herein demonstrated for the first time that, although NPCs are capable of engulfing *P. acnes* and forming phagosomes to subsequently eliminate *P. acnes* with the participation of mROS, the fact remains that *P. acnes* can evade microbicidal phagocytosis by inhibiting the mitochondrial biogenesis of nucleus pulposus cells. This allows *P. acnes* to reside in intervertebral discs for an extended period, thereby inducing IVDD. These findings provide novel insights into how *P. acnes* avoids being phagocyted and killed by NPCs and suggest potentially new targets for treating disc infections.

## Results

### *P. acnes* colonizes within a portion of degenerated NP tissues

**Fig. 1:**
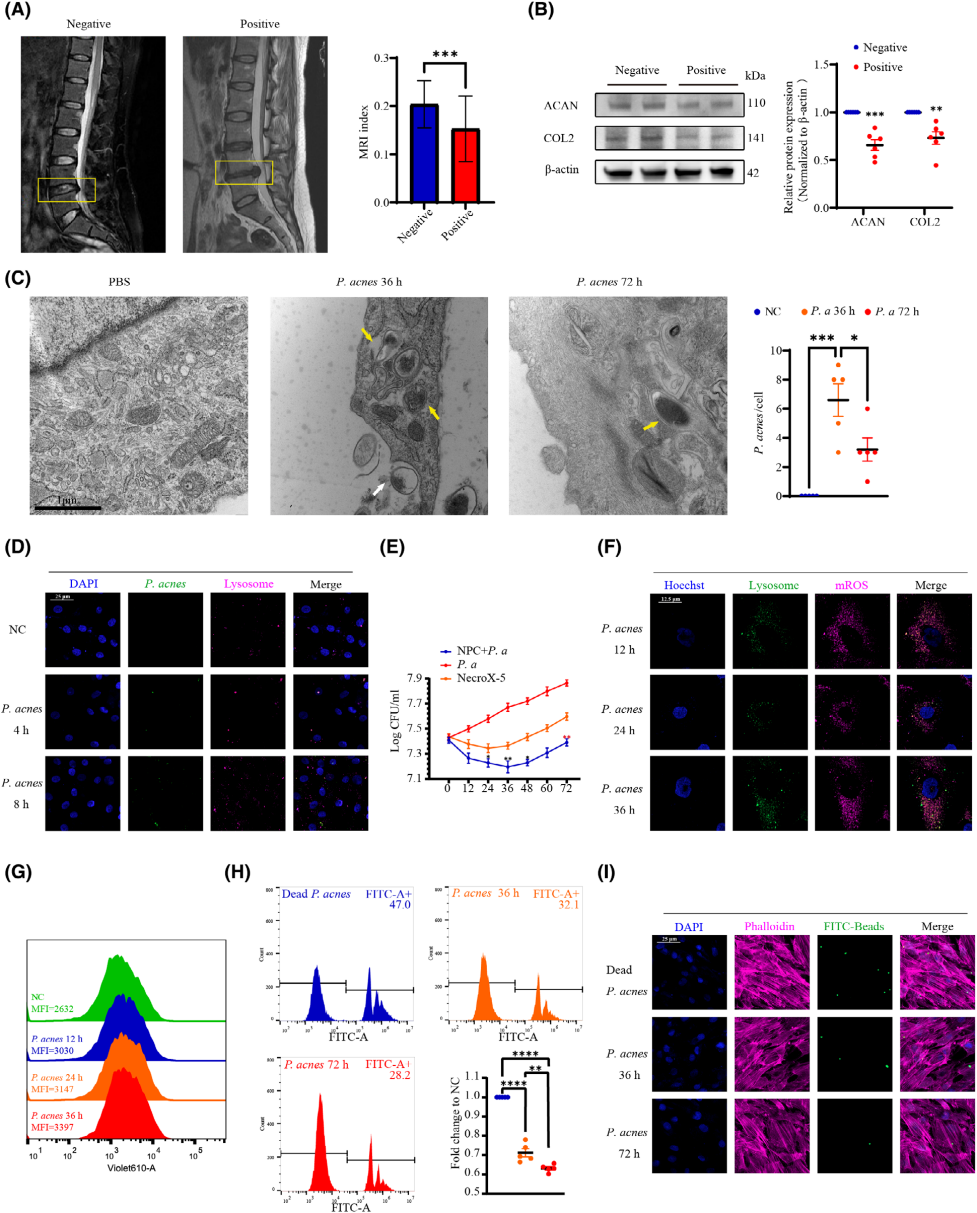
*P. acnes* colonizes a portion of degenerated NP tissues and promotes its long‐term colonization by inhibiting the sustained microbicidal function of NPCs. (A) Representative images from MRI point to severe degeneration in *P. acnes*‐positive IVDs (*N* = 69 for the negative group, *N* = 18 for the positive group; *P* values were analyzed using Student's *t* test, and data are presented as the mean ± SD). (B) Western blot analysis of aggrecan and collagen II expression (*N* = 6). (C) Electron microscopic images of NPCs phagocytizing *P. acnes*. Ingested *P. acnes* (yellow arrows) in the phagosome within the cytoplasm of NPCs and membrane protrusions tightly engulfing *P. acnes* (white arrows) was observed (Scale bar: 1 μm). (D) Staining of LysoTracker Red in NPCs infected with *P. acnes* labeled by BacLight for 4 and 8 h (Scale bar: 25 μm). (E) The survival of extracellular *P. acnes* was determined by plate counting. The groups at 24, 36, and 48 h were significantly different from the group at 0 h, and the groups at 72 h were significantly different from the groups at 36 h. (F) Staining of LysoTracker Green and MitoSox in NPCs infected with *P. acnes* for 12, 24, and 36 h (Scale bar: 12.5 μm). (G) Staining of MitoSox in NPCs infected with *P. acnes* for 12, 24, and 36 h; the total cellular mROS was analyzed by flowcytometry. (H) NPCs were pretreated with active or inactive *P. acnes* for 36 and 72 h and incubated with latex beads for 8 h, after which the interaction was compared by flow cytometric analysis. (I) NPCs pretreated with active or inactive *P. acnes* for different periods were analyzed by confocal microscopy after incubation with latex beads (Scale bar: 25 μm). *N* = 5, *P < 0.05, **P < 0.01, ***P < 0.001, ****P < 0.0001; values were analyzed using Student's *t* test or one‐way ANOVA; data are presented as the mean ± SEM. COL2, collagen II; IVDs, intervertebral discs; mROS, mitochondrial reactive oxygen species; NP, nucleus pulposus; NPCs, nucleus pulposus cells; *P. acnes/P. a*, *Propionibacterium acnes*.

To investigate the relationship between *P. acnes* colonization and chronic intervertebral disc degeneration, we collected 87 degenerated discs from patients and found 18 (20.69%) to be positive for *P. acnes*, consistent with our previous findings (21.70%). Compared with the *P. acnes*‐negative group, the mean age of the *P. acnes*‐positive group was significantly lower, and the Pfirrmann grade (indicating the degree of degeneration) was significantly higher (Table 1). There were no differences in terms of sex or affected spinal segments between the two groups. When we conducted a quantitative analysis of MRI indices and determined the protein expression of aggrecan and collagen II to assess the degenerative status of the different groups, our results revealed a significant drop in the MRI index and protein expression in the *P. acnes*‐positive group (Fig. 1A,B). These findings confirmed the presence of *P. acnes* colonization in the NP tissue of patients with chronic disc degeneration, implying that *P. acnes* infection may have contributed to the initiation and/or worsening of IVDD.

**Table 1:** Characteristics of patients with intervertebral disc degeneration.

	*P. acnes*‐positive	*P. acnes*‐negative	*P*‐value
The number of patients	18	69	
Age (years)	34.72 ± 12.41	40.28 ± 9.73	0.045*
Gender			
Male	9	36	0.999
Female	9	33	
Diseased segment (*n*)			
L4/5	6	29	0.595
L5/S1	12	40	
Pfirrmann grade (*n*) (II/III/IV/V)	2/5/8/3	9/42/15/3	0.031*

*P < 0.05.

### *P. acnes* promotes its long‐term colonization by inhibiting the sustained bactericidal function of NPCs

Our previous study confirmed the microbicidal activity of NPCs against *Staphylococcus aureus* through the induction of phagolysosome formation [20]. We next sought to investigate the mechanism by which the bactericidal function of NPCs fails to completely clear the colonization of *P. acnes* in NP tissues and leads to degenerative changes associated with chronic infection.

We first evaluated the ingestion of *P. acnes* by NPCs using TEM, and after co‐culturing the NPCs with *P. acnes* for 36 h, we observed ingested *P. acnes* in the phagosome within the cytoplasm of NPCs (indicated by yellow arrows in Fig. 1C) and membranous protrusions that tightly engulfed *P. acnes* (indicated by white arrows in Fig. 1C). This indicated that NPCs were able to ingest and internalize *P. acnes*.

The ingestion of bacteria by professional phagocytes typically leads to the fusion of phagosomes with primary lysosomes, resulting in the formation of phagolysosomes [21]—a process that facilitates the killing of pathogens and disrupting microbial components. To determine whether NPCs induced this fusion to eliminate ingested *P. acnes* bacteria like professional phagocytes, we evaluated the formation of secondary lysosomes using LysoTracker Red (Molecular Probes), a fluorescent marker for late endosomes and lysosomes, and we assessed the maturation of phagosomes containing *P. acnes* by tracking their co‐localization with LysoTracker Red (Fig. 1D). Our results indicated a gradual increase in the number of cells positive for LysoTracker Red and ingested *P. acnes* over the incubation period (0–8 h), along with an elevated percentage of co‐localization. As phagolysosome formation progressed, enumeration of bacterial colonies showed that viable *P. acnes* was progressively eradicated by NPCs in a time‐dependent manner from 0 to 36 h (Fig. 1E). Previous reports indicated that the clearance of pathogenic microorganisms by phagosomes depends on mROS delivered by mitochondria‐derived vesicles [22]. We utilized NecroX‐5, a mROS scavenging agent, to demonstrate that NPC clearance of intracellular *P. acnes* depends on mROS (Fig. 1E).

However, as the co‐cultivation time was extended to 48–72 h, we recognized a more intriguing phenomenon; that is, a decrease in the number of intracellular *P. acnes* and an increase in the number of extracellular *P. acnes*, regardless of whether the mROS of NPC was cleared or not (Fig. 1C,E).

We then asked whether the existence of *P. acnes* further inhibited the microbicidal function of the NPCs. To verify this, we first detected the co‐localization of mROS and lysosomes, and we found that compared to the control group, which was stimulated with inactivated *P. acnes*, the live *P. acnes* reduced the ratio of mROS co‐localization with lysosomes (Fig. 1F), rather than by decreasing the total amount of mROS in the NPCs (Fig. 1G), which indicates that the infection of *P. acnes* affects the ability of mitochondria to deliver mROS to lysosomes. Meanwhile, the increased mROS also indicates that *P. acnes* may impair the mitochondria of NPCs through other mechanisms.

Subsequently, we examined the phagocytic ability of NPCs pretreated with *P. acnes* for different lengths of time on latex beads, as previous studies have reported that NPCs can effectively phagocytose latex beads [23]. As shown in Fig. 1H, live *P. acnes* inhibited the phagocytosis of NPCs on latex beads in a time‐dependent manner compared with the control group, with a clear inhibition observed at 72 h compared with 36 h. Furthermore, confocal fluorescence microscopy exhibited a significant decline in the number of latex beads in the cytoplasmic area of NPCs stimulated with *P. acnes* for 72 h compared with 36 h (Fig. 1H). Overall, these data showed the bactericidal function of the NPCs against *P. acnes*, while also suggesting that the existence of *P. acnes* further inhibited the antimicrobial effect of the NPCs.

### *P. acnes* inhibits sustained microbicidal function of NPCs by suppressing their mitochondrial biogenesis

**Fig. 2:**
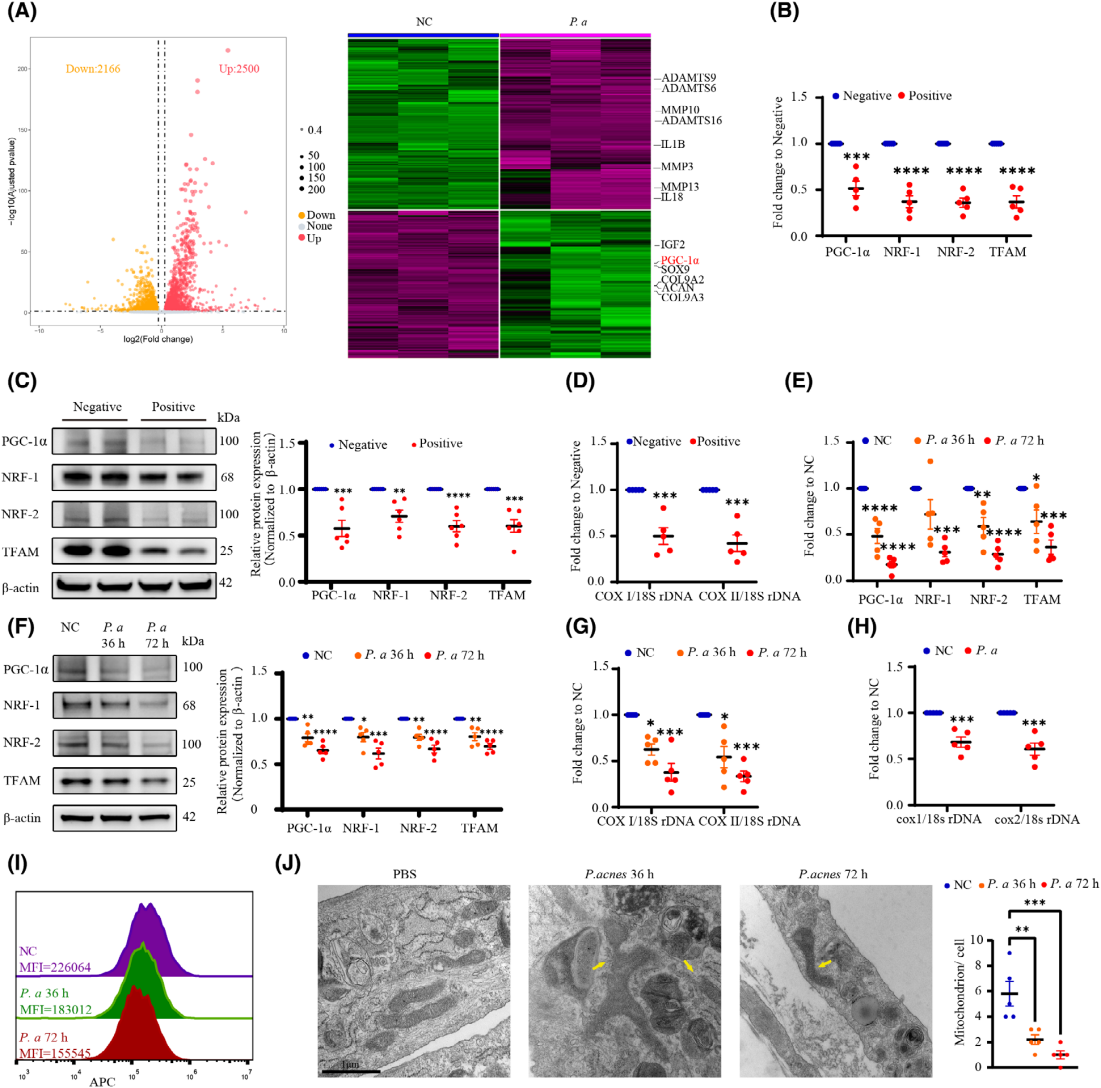
*P. acnes* reduces mitochondrial biogenesis in NPCs. (A) DEGs in *P. acnes*‐negative or ‐positive samples (*N* = 3). (B) Quantification of mRNA expression. (C) Western blot analysis of PGC‐1a, NRF1/2, and TFAM (*N* = 6). (D) MtDNA content as determined by the mtDNA‐encoded COX1 or COX2: nucleus‐encoded 18S rDNA ratio. (E and F) Quantification of mRNA expression (E) and western blot analysis (F) of PGC‐1a, NRF1/2, and TFAM in NPCs at 36 and 72 h post‐infection. (G) NPCs were infected with *P. acnes* at an MOI of 100 for 36 and 72 h; mitochondrial DNA content as determined by the COX1:18S rDNA or COX2:18S rDNA ratio. (H) Mitochondrial DNA content of rat caudal IVDs inoculated with *P. acnes*. (I) Fluorescence histogram of mitochondrial mass by flow‐cytometric analysis. Mitochondria of cells were labeled with MitoTracker Deep Red probes and assayed at 36 and 72 h post‐infection. (J) Electron microscopic images of mitochondria. NPCs infected with *P. acnes* at an MOI of 100 were examined at 36 and 72 h post‐infection. The quality of the mitochondria in NPCs was significantly impaired (yellow arrows). We analyzed the number of mitochondria per cell statistically (Scale bar: 1 μm). *N* = 5, *P < 0.05, **P < 0.01, ***P < 0.001, ****P < 0.0001; values were analyzed using Student's *t* test or one‐way ANOVA; data are presented as the mean ± SEM. 18S rDNA, 18S ribosomal DNA; COX1, cytochrome c oxidase subunit 1; COX2, cytochrome c oxidase subunit 2; DEGs, differentially expressed genes; IVDs, intervertebral discs; MOI, multiplicity of infection; mtDNA, mitochondrial DNA; nDNA, nuclear DNA; NPCs, nucleus pulposus cells; NRF1, nuclear respiratory factor 1; NRF2, nuclear factor erythroid 2‐related factor 2; *P. acnes/P. a*, *Propionibacterium acnes*; PGC‐1α, peroxisome proliferator‐activated receptor gamma coactivator‐alpha; TFAM, transcription factor A, mitochondrial.

To investigate the mechanisms underlying dysfunctional bacterial killing induced by *P. acnes*, we implemented mRNA‐seq experiments. Among the significantly and differentially expressed mRNAs, 2166 genes were down‐regulated (< 1.2‐fold, *P* < 0.05), and 2500 genes were upregulated (> 1.2‐fold, *P* < 0.05) in the *P. acnes*‐positive group relative to the negative group (Fig. 2A). Consistent with previous reports, mRNA‐seq revealed several differentially expressed genes (DEGs) correlated with IVDD, including SOX9, ACAN, MMP13, IGF2, and IL1B, indicating that chronic *P. acnes* infections induced IVDD. Additionally, down‐regulated PGC‐1α attracted our attention; PGC‐1α served as the ‘master regulator’ of mitochondrial biogenesis and was significantly suppressed in *P. acnes*‐positive discs.

Mitochondrial biogenesis is a key mechanism that maintains mitochondrial homeostasis, and disruption of mitochondrial homeostasis inhibits the delivery of mROS and phagocytosis function [24, 25, 26]. We therefore sought to verify whether *P. acnes* impaired mitochondrial biogenesis and analyzed the mRNA and protein expression for PGC‐1α (the critical factor involved) and downstream factors (NRF1/2 and TFAM), and discerned that the relative expression in the *P. acnes*‐positive group was much lower than in the *P. acnes*‐negative group (Fig. 2B,C). In addition, a decreased ratio of mitochondrial DNA (mtDNA)/nuclear DNA (nDNA) was found in *P. acnes*‐positive samples (Fig. 2D). Collectively, it was reasonable to hypothesize that *P. acnes* impaired mitochondrial biogenesis.

To further verify the above hypothesis, we constructed a system of co‐culturing NPCs and *P. acnes* at a 100 : 1 multiplicity of infection (MOI) for different time periods (36 and 72 h), and observed that the mRNA expression for PGC‐1α, NRF1/2, and TFAM was also reduced in a time‐dependent manner (Fig. 2E), similar to our results with western blotting (Fig. 2F). The NPC mtDNA/nDNA ratio also declined significantly (Fig. 2G), consistent with the data from our *in vivo* experiments (Fig. 2H), indicating a decrease in mitochondrial numbers. Flow‐cytometric analysis using MitoTracker Deep Red staining showed that the mean fluorescence intensity of NPCs gradually fell with time in coculture (Fig. 2I). In addition, when we examined NPCs post‐infected by *P. acnes* using TEM, we noted that the quality of the mitochondria in NPCs was significantly impaired (Fig. 2J), signified by irregular shapes, disrupted bilateral membrane structures, a diminution in mitochondrial cristae, and marked reduction in the number of mitochondria per NPC. These results suggested that *P. acnes* infection impaired mitochondrial biogenesis in NPCs.

**Fig. 3:**
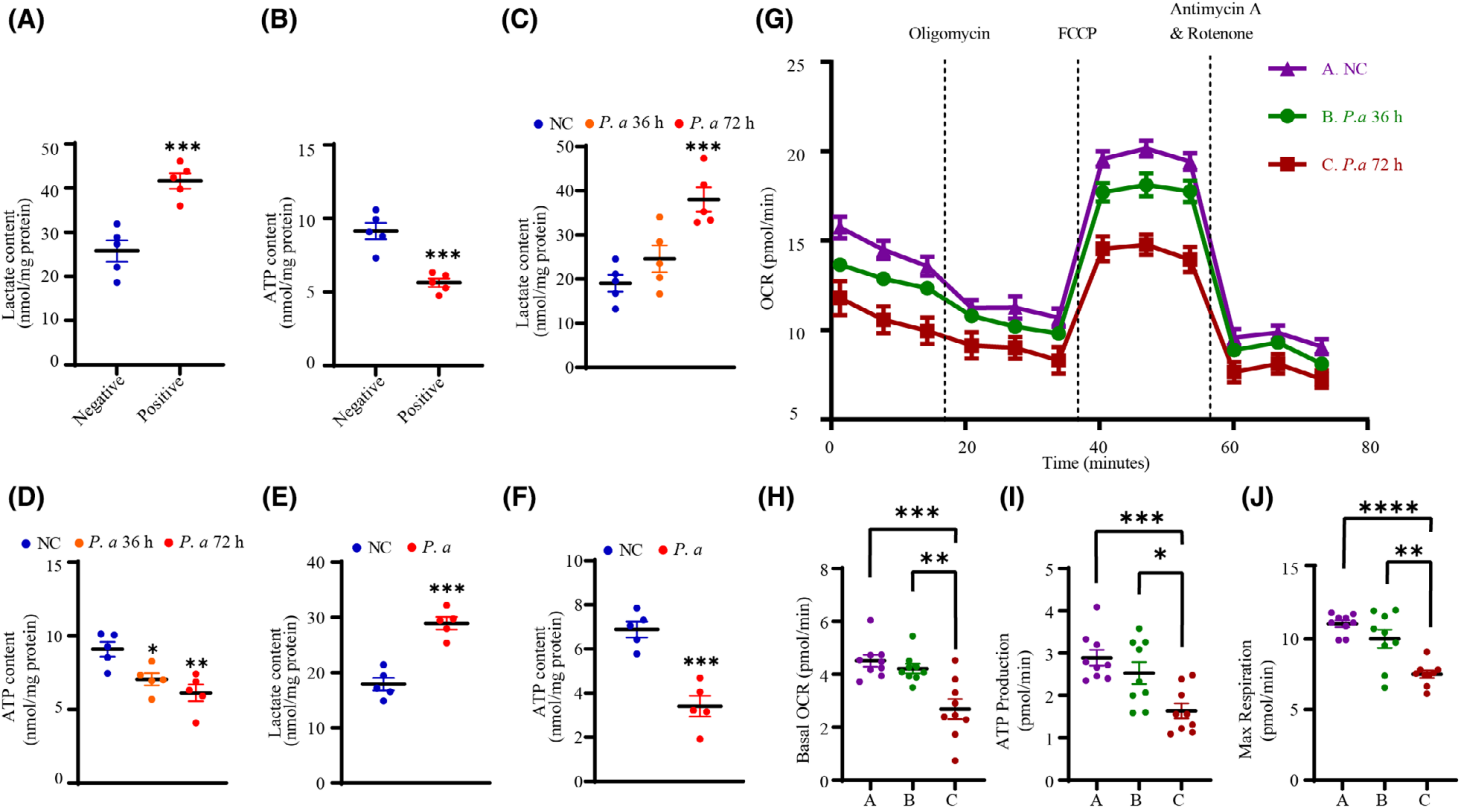
*P. acnes* induces a diminution in mitochondrial respiration. (A and B) Lactate and ATP content in NP tissues. (C and D) Lactate and ATP content in NPCs after incubation with *P. acnes* for different periods at an MOI of 100. (E and F) Lactate and ATP content in rat caudal IVDs infected with *P. acnes*. (G–J) OCR profiles (G), changes in basal OCR (H), changes in ATP production (I), and changes in maximal respiration (J) of NPCs after incubation with *P. acnes* for different periods at an MOI of 100 (*N* = 9). *N* = 5, *P < 0.05, **P < 0.01, ***P < 0.001, ****P < 0.0001; values were analyzed using Student's *t* test or one‐way ANOVA; data are presented as the mean ± SEM. IVDs, intervertebral discs; MOI, multiplicity of infection; NP, nucleus pulposus; NPCs, nucleus pulposus cells; OCR, oxygen‐consumption rate; *P. acnes/P. a*, *Propionibacterium acnes*.

Impairment of mitochondrial biogenesis might inhibit the oxidative metabolism of cells and thus disrupt cellular homeostasis. To test this, we determined the concentrations of lactic acid and ATP in the disc tissues from different groups. In positive tissues, lactic acid rose dramatically, implying an increase in glycolysis within NPCs. ATP content also declined noticeably, suggesting a diminution in oxidative metabolism (Fig. 3A,B), with results identical to those in the co‐culturing system (Fig. 3C,D) and in rats *in vivo* (Fig. 3E,F). We subsequently evaluated mitochondrial function by a Seahorse XFe96 analyzer and observed that the basal oxygen‐consumption rate (OCR) of the NPCs declined commensurate with coculture time (Fig. 3G,H). Concomitantly, *P. acnes* inhibited ATP production and max OCR (Fig. 3I,J), indicative of impairment in the content or electron transport activity of mitochondria. These results indicated that oxidative metabolism was impaired by *P. acnes*.

We know that a switch in energy production from mitochondrial respiration to glycolysis would disrupt cellular phagocytotic function [27]. After observing decreased levels of PGC‐1α and impaired mitochondrial respiration—as well as reduced microbicidal phagocytosis of NPCs induced by *P. acnes*—we investigated whether restoration of mitochondrial biogenesis via PGC‐1α overexpression would reverse the attenuated phagocytosis elicited by *P. acnes*.

**Fig. 4:**
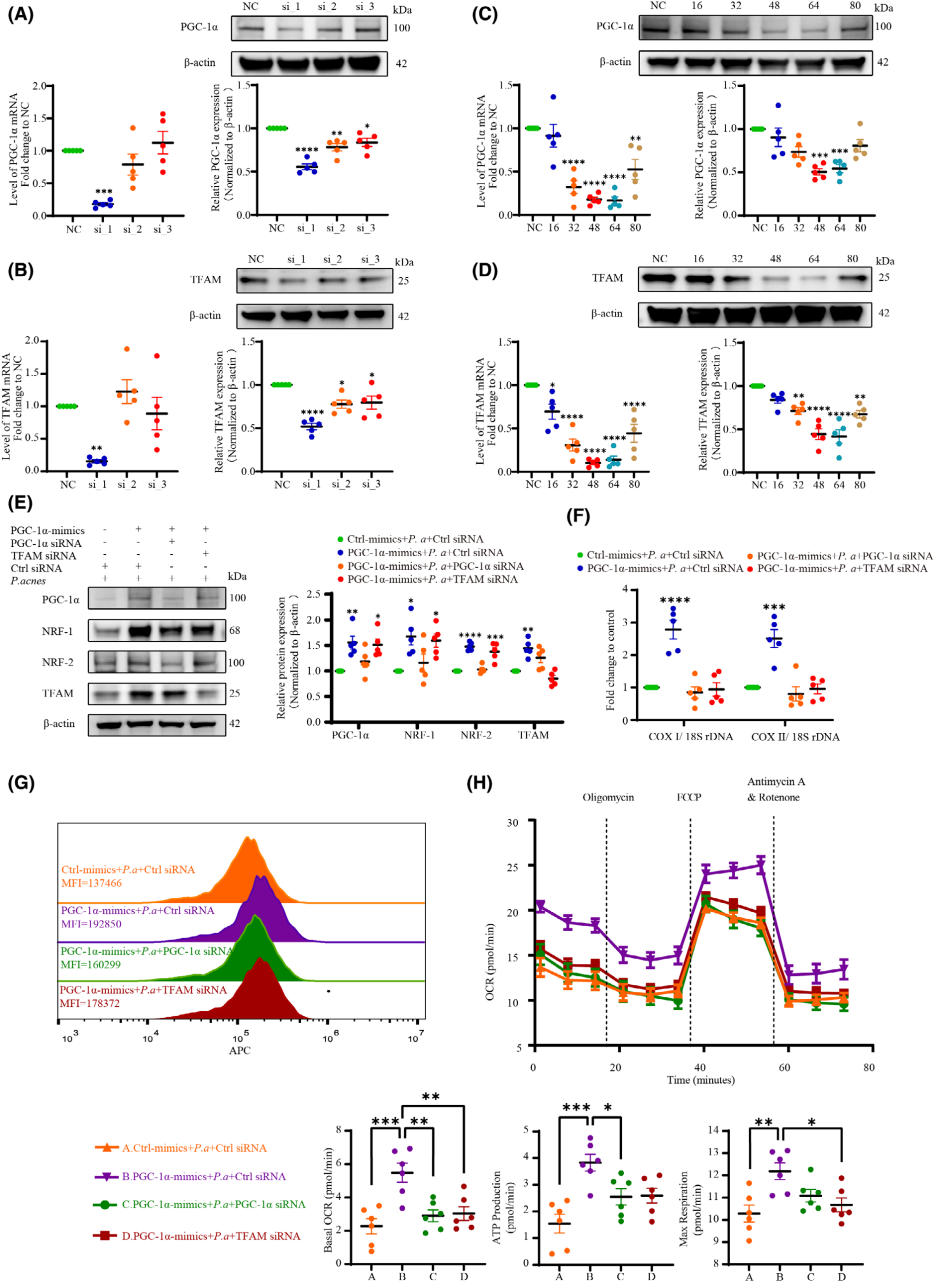
PGC‐1α overexpression restores mitochondrial biogenesis and respiration impaired by *P. acnes*. (A) The expression of mRNA and protein corresponding to the knockdown of PGC‐1α using different siRNAs. (B) The expression of mRNA and protein corresponding to the knockdown of TFAM using different siRNAs. (C) The expression curves of mRNA and protein of PGC‐1α over time after silencing PGC‐1α with siRNA. (D) The expression curves of mRNA and protein of TFAM over time after silencing TFAM with siRNA. (E) Western blot and quantitative analyses of PGC‐1α, NRF1/2, and TFAM. NPCs were pre‐transfected with or without lentiviral vector and siRNA and then infected with *P. acnes* for 72 h. (F) Mitochondrial DNA content as determined by the COX1:18S rDNA or COX2:18S rDNA ratio. NPCs were pre‐transfected with or without lentiviral vector and siRNA, and then infected with *P. acnes* for 72 h. (G) Fluorescence histogram of mitochondrial mass by flow‐cytometric analysis. NPCs were pre‐transfected with or without lentiviral vector and siRNA, and then infected with *P. acnes* for 72 h. The mitochondria of cells were labeled with MitoTracker Deep Red probes. (H) OCR profiles, changes in basal OCR, ATP production, and maximal respiration of NPCs after pre‐transfection with or without lentiviral vector and siRNA before incubating with *P. acnes* for 72 h (*N* = 6). *N* = 5, *P < 0.05, **P < 0.01, ***P < 0.001, ****P < 0.0001; values were analyzed using one‐way ANOVA; data are presented as the mean ± SEM. NPCs, nucleus pulposus cells; OCR, oxygen‐consumption rate; *P. acnes/P. a*, *Propionibacterium acnes*.

First, we designed three distinct siRNAs targeting PGC‐1α and TFAM, respectively, and evaluated their knockdown efficiency using qPCR and western blotting (Fig. 4A,B). Next, we verified the expression curves of PGC‐1α and TFAM over time following siRNA transfection, confirming that the designed siRNAs exhibited highly significant knockdown effects between 48 and 64 h post‐transfection (Fig. 4C,D). Subsequently, NPCs were treated with either a PGC‐1α overexpression lentivirus, PGC‐1α/TFAM siRNA, or *P. acnes* to elucidate the roles of PGC‐1α and TFAM in mitochondrial damage induced by *P. acnes*. Our results, as shown in Fig. 4E, revealed that PGC‐1α overexpression significantly augmented levels of mitochondrial biogenesis‐related proteins, such as NRF‐1, NRF‐2, and TFAM, and mitochondrial copy number (Fig. 4F), as indicated by the ratio of cytochrome c oxidase subunit 1 (COX1) and cytochrome c oxidase subunit 2 (COX2) DNA copies to 18S ribosomal DNA (18S rDNA). Mitochondrial quality and respiratory capability were also enhanced in the presence of PGC‐1α overexpression. However, these processes were significantly inhibited by knocking down PGC‐1α or TFAM (Fig. 4G,H).

**Fig. 5:**
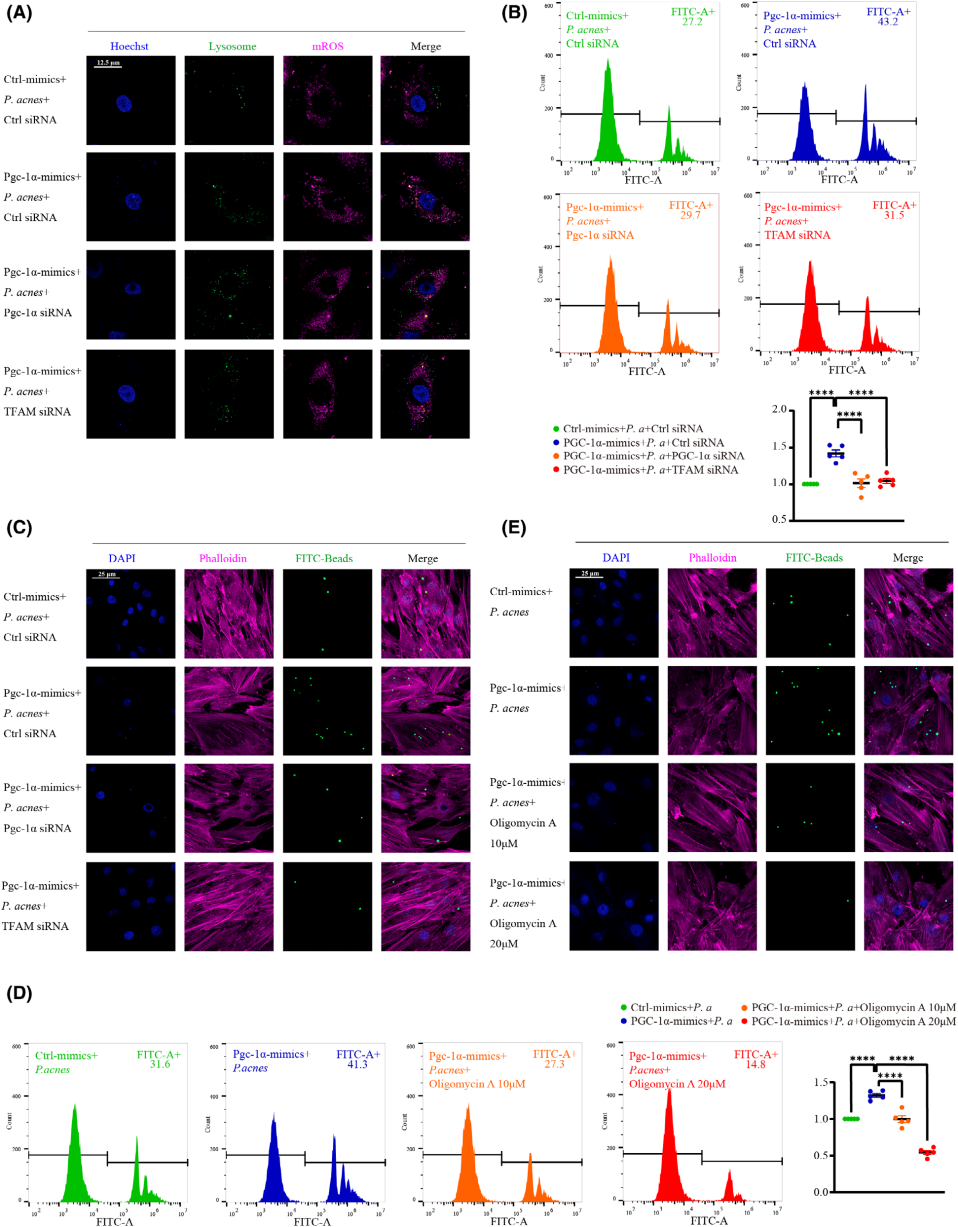
*P. acnes* inhibits the sustained microbicidal ability of NPCs by suppressing their mROS delivery and ATP production via dampening mitochondrial biogenesis. (A) Staining of LysoTracker Green and MitoSox in NPCs infected with *P. acnes*, NPCs were transfected with or without lentiviral vector and siRNA (Scale bar: 12.5 μm). (B and C) NPCs were pre‐transfected with or without lentiviral vector and siRNA, infected with *P. acnes* for 72 h, and incubated with latex beads for 8 h. The interactions were compared by flow‐cytometric analysis (B), and confocal microscopy (C) (Scale bar: 25 μm). (D and E) NPCs were pre‐transfected with lentiviral vector, infected with *P. acnes* for 72 h, and incubated with latex beads and oligomycin A (10 or 20 μm) for 8 h. The interactions were compared by flow‐cytometric analysis (D) and confocal microscopy (E) (Scale bar: 25 μm). *N* = 5, ****P < 0.0001; values were analyzed using one‐way ANOVA; data are presented as the mean ± SEM. NPCs, nucleus pulposus cells; *P. acnes/P. a*, *Propionibacterium acnes*.

After confirming that overexpression of PGC‐1α significantly promoted mitochondrial biogenesis and mitochondrial respiration in response to *P. acnes*, we further examined whether the bactericidal function of NPCs was also enhanced. As shown in Fig. 5A, overexpression of PGC‐1α rescues impaired mitochondrial mass and significantly improves the ratio of mROS and lysosome co‐localization, which represents the restoration of lysosomal microbicidal function. At the same time, overexpression of PGC‐1α significantly increased the internalization of latex beads by NPCs pretreated with *P. acnes*, as indicated by the number of latex beads inside the NPCs, while knockout of PGC‐1α or TFAM significantly inhibited this process (Fig. 5B,C). In addition, the promotion of phagocytosis by overexpression of PGC‐1α depended on mitochondrial respiration, and oligomycin A, an ATP synthase inhibitor that disrupts the coupling of mitochondrial membranes, inhibited oxidative phosphorylation and all ATP‐dependent reactions, and oligomycin A significantly inhibited the internalization of latex beads (Fig. 5D,E).

These findings collectively demonstrated that promoting mitochondrial biogenesis increased the proportion of mitochondrial delivery of mROS to lysosomes, enhanced the bactericidal function of NPC, and also enhanced mitochondrial respiration to produce more ATP to engulf pathogens, while mitochondrial biogenesis and mitochondrial respiration impairment reduced the bactericidal function of NPC against *P. acnes*.

**Fig. 6:**
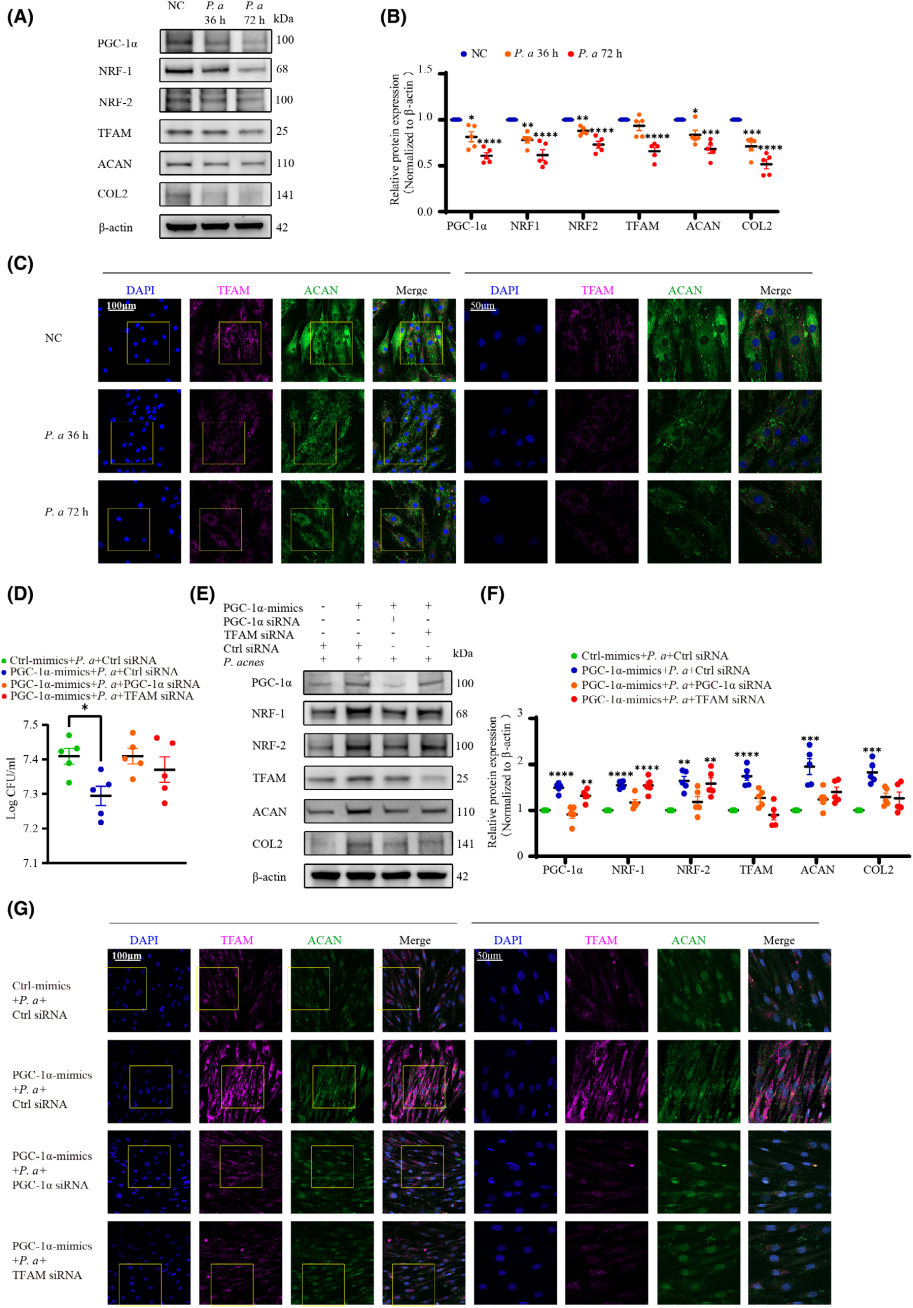
Enhancing mitochondrial biogenesis reduces the onset of intervertebral disc degeneration by improving the phagocytic function of NPCs *in vitro*. (A and B) Western blot (A) and quantitative analyses (B) of mitochondrial biogenesis‐related factors, aggrecan, and collagen II in NPCs at 36 and 72 h post‐infection. (C) Immunofluorescence analysis of TFAM and aggrecan in NPCs in response to *P. acnes* infection at 36 and 72 h post‐infection; the right panels show magnified insets (Scale bar: 100/50 μm). (D) Survival of extracellular *P. acnes* was determined by plate counting. (E and F) Western blot (E) and quantitative analyses (F) of mitochondrial biogenesis‐related factors, aggrecan, and collagen II. NPCs were pre‐transfected with or without lentiviral vector and siRNA, and then infected with *P. acnes* for 72 h. (G) Immunofluorescence analysis of TFAM and aggrecan in NPCs after pre‐transfection with or without lentiviral vector and siRNA before incubating with *P. acnes* for 72 h; the right panels show magnified insets (Scale bar: 100/50 μm). *N* = 5, *P < 0.05, **P < 0.01, ***P < 0.001, ****P < 0.0001; values were analyzed using one‐way ANOVA; data are presented as the mean ± SEM. NPCs, nucleus pulposus cells; *P. acnes/P. a*, *Propionibacterium acnes*.

Our previous studies confirmed that the colonization of *P. acnes* led to a reduction in aggrecan and collagen II, indicating the onset of NPC degeneration, and we observed this phenomenon once again *in vitro* (Fig. 6A–C). We next explored whether enhancing mitochondrial biogenesis and mitochondrial respiration would reduce the onset of IVDD by improving the phagocytic function of NPCs *in vitro*. Bacterial colony‐counting results revealed that the activation of mitochondrial biogenesis significantly enhanced the bacterial clearance function of NPCs and that this effect was hindered by PGC‐1α/TFAM knockdown (Fig. 6D). Furthermore, western blot analysis showed that NPC degeneration was reduced after upregulation of mitochondrial biogenesis and inhibition of *P. acnes* colonization (Fig. 6E,F), consistent with our laser confocal finding (Fig. 6G). When PGC‐1α/TFAM was knocked down, the ability of NPCs to clear *P. acnes* was reduced and NPC degeneration occurred.

**Fig. 7:**
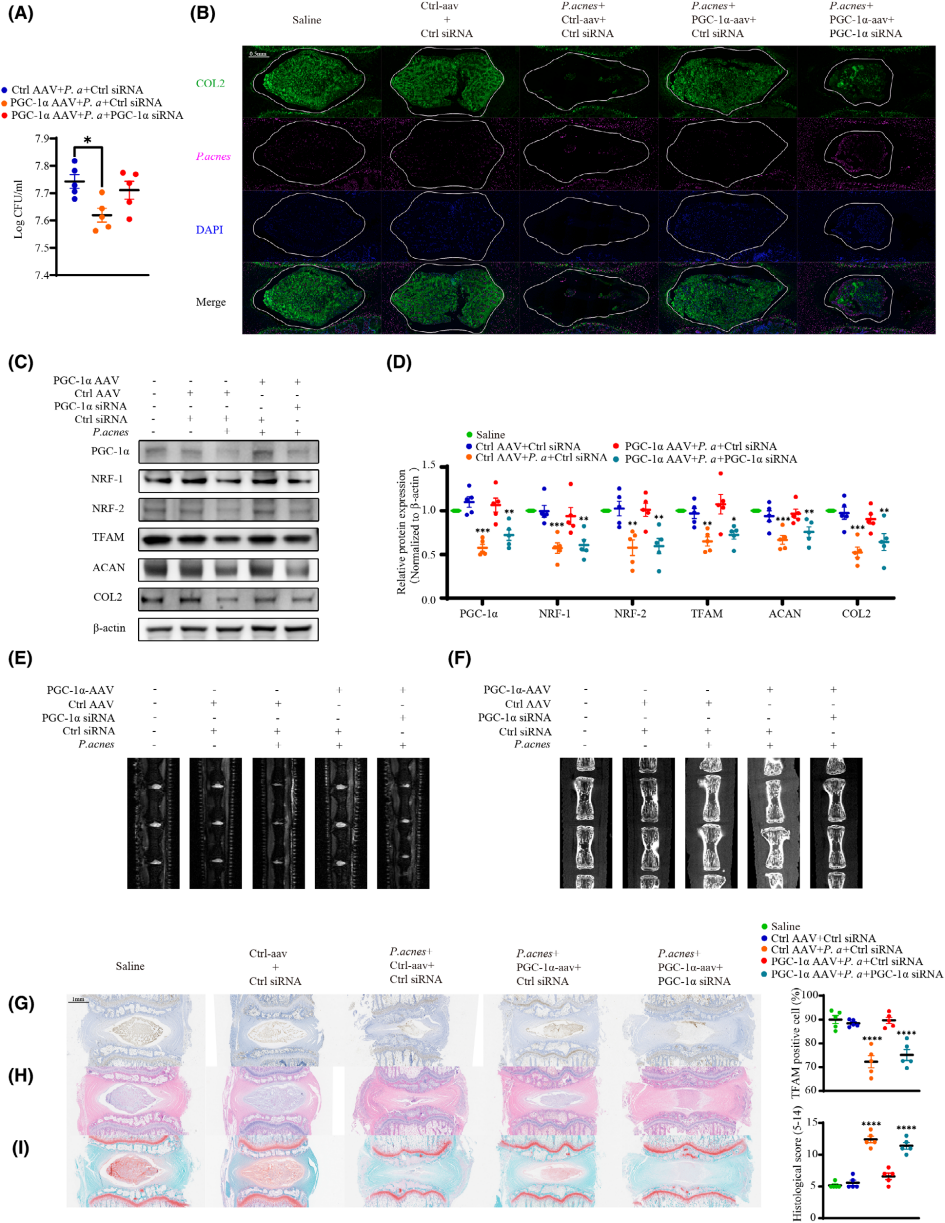
Enhancing mitochondrial biogenesis reduces the occurrence of IVDD by improving the phagocytic function of NPCs *in vivo*. (A) Survival of extracellular *P. acnes* was determined by plate counting. (B) Immunofluorescence analysis of *P. acnes* and collagen II in rat caudal discs inoculated with or without aav, siRNA, and *P. acnes*; the NP tissue was marked with white lines (Scale bar: 0.5 mm). (C and D) Western‐blot (C) and quantitative analyses (D) of mitochondrial biogenesis‐related factors, aggrecan, and collagen II in rat caudal IVDs inoculated with or without aav, siRNA, or *P. acnes*. (E) MRI analysis of caudal discs. (F) Micro‐CT analysis of caudal discs. (G) IHC staining for TFAM and statistical graphs of the percentage of TFAM‐positive cells in rat caudal IVDs (Scale bar: 1 mm). (H and I) HE staining (H), safranin O staining (I), and statistical graphs of histological scores in serial sections of rat caudal IVDs (Scale bar: 1 mm). *N* = 5, *P < 0.05, **P < 0.01, ***P < 0.001, ****P < 0.0001; values were analyzed using one‐way ANOVA; data are presented as the mean ± SEM. IVDD, intervertebral disc degeneration; IVDs, intervertebral discs; NP, nucleus pulposus; NPCs, nucleus pulposus cells; *P. acnes/P. a*, *Propionibacterium acnes*.

To examine the effects of PGC‐1α on phagocytosis *in vivo*, we infected rat caudal IVDs with PGC‐1α‐overexpressing adeno‐associated virus. Two weeks after surgery, *P. acnes* and PGC‐1α siRNA were inoculated into the caudal IVDs of rats, and we observed from the *P. acnes* infection model of NP tissue that restoration of PGC‐1α expression in the NP effectively prevented the colonization of *P. acnes* and NP degeneration (Fig. 7A,B). This beneficial effect was significantly hindered by the PGC‐1α knockdown. Furthermore, the restoration of PGC‐1α expression led to an increase in the expression of NRF‐1, NRF‐2, and TFAM in NP tissue, as well as a notable rise in the levels of aggrecan and collagen II (Fig. 7C,D). These findings were supported by results from MRI and CT modalities, as well as by IHC, HE, and safranin O staining (Fig. 7E–I), confirming the protective role of restoring PGC‐1α expression against *P. acnes*‐induced NP degeneration. However, these processes were substantially impaired by the knockdown of PGC‐1α. In conclusion, our study demonstrated that enhancing mitochondrial biogenesis and mitochondrial respiration mitigated infectious IVDD by improving the phagocytic function of NPCs.

### *P. acnes* impairs mitochondrial biogenesis of NPCs via the AMPK/SIRT‐1/PGC‐1α signaling pathway

**Fig. 8:**
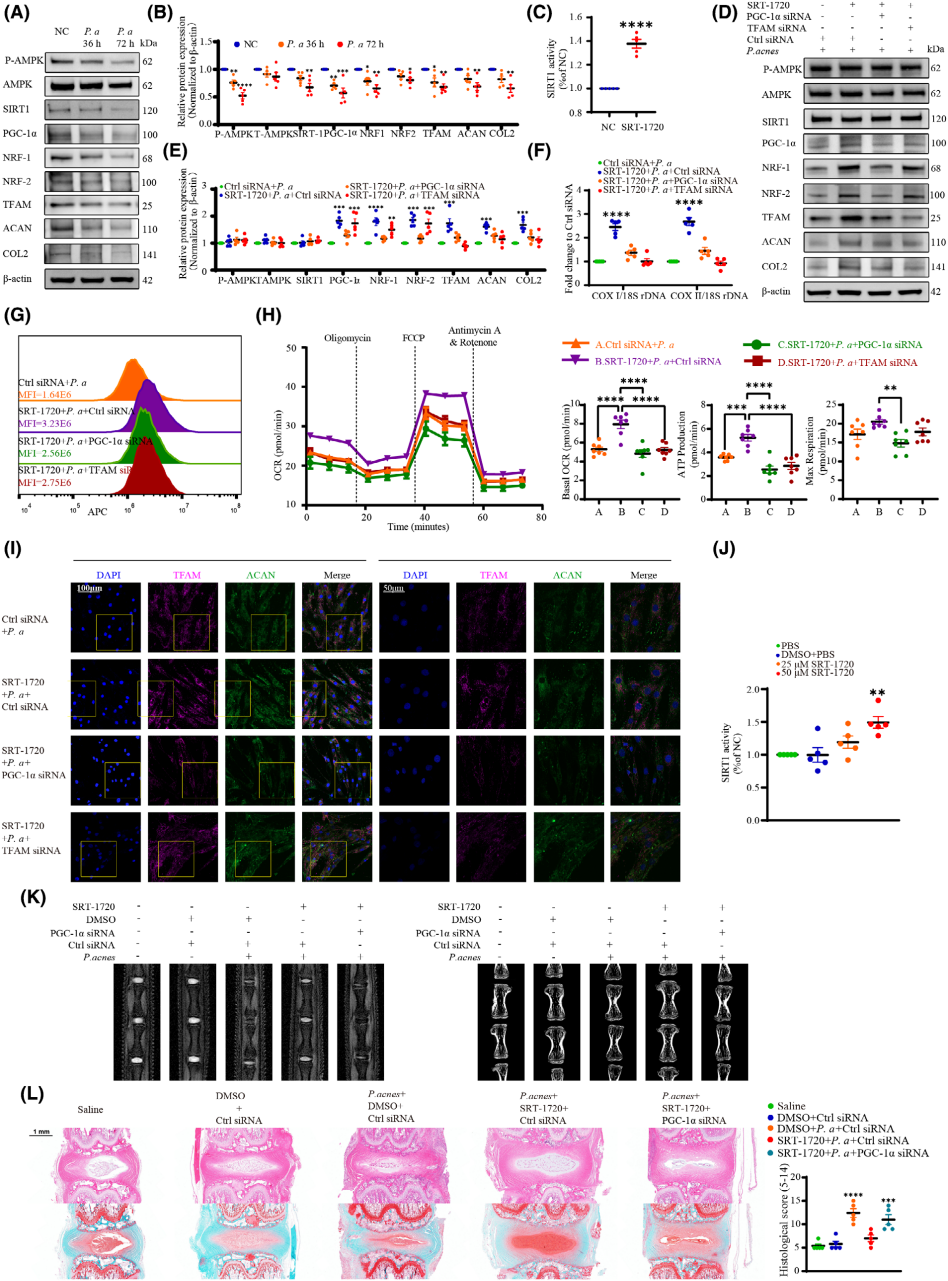
*P. acnes* impairs mitochondrial biogenesis by inhibiting SIRT1. (A and B) Western blot (A) and quantitative analyses (B) of p‐AMPK, total AMPK, SIRT‐1, mitochondrial biogenesis‐related factors, aggrecan, and collagen II in NPCs at 36 and 72 h post‐infection. (C) SIRT‐1 activity in NPCs stimulated with SRT‐1720 (5 μm). (D and E) Western blot (D) and quantitative analyses (E) of p‐AMPK, total AMPK, SIRT‐1, mitochondrial biogenesis‐related factors, aggrecan, and collagen II. NPCs were pretreated with or without SRT‐1720 (5 μm) and siRNA, and then infected with *P. acnes* for 72 h. (F and G) Mitochondrial DNA content and (F) fluorescence histogram of mitochondrial mass (G) in NPCs after pretreatment with or without SRT‐1720 (5 μm) and siRNA before incubation with *P. acnes* for 144 h. (H) OCR profiles, changes in basal OCR, ATP production, and maximal respiration of NPCs after pretreatment with or without SRT‐1720 (5 μm) or siRNA before incubation with *P. acnes* for 72 h (*N* = 7). (I) Immunofluorescence analysis of TFAM and aggrecan in NPCs. NPCs were pretreated with or without SRT‐1720 (5 μm) and siRNA, and then infected with *P. acnes* for 72 h; the right panels show magnified insets (Scale bar: 100/50 μm). (J) SIRT‐1 activity in NP following intradiscal administration of SRT‐1720 in rats. (K) MRI analysis and Micro‐CT analysis of caudal discs. (L) HE staining, safranin O staining, and statistical graphs of histological scores in serial sections of rat caudal IVDs (Scale bar: 1 mm). *N* = 5, *P < 0.05, **P < 0.01, ***P < 0.001, ****P < 0.0001; values were analyzed using one‐way ANOVA; data are presented as the mean ± SEM. AMPK, AMP‐activated protein kinase; IVDs, intervertebral discs; NP, nucleus pulposus; NPCs, nucleus pulposus cells; OCR, oxygen‐consumption rate; *P. acnes/P. a*, *Propionibacterium acnes*; SIRT‐1, sirtuin 1.

We then explored the molecular mechanism involved in *P. acnes*‐induced mitochondrial biogenesis impairment of NPCs. Since the AMPK/SIRT‐1/PGC‐1α pathway is a well‐known signaling system for mitochondrial homeostasis [24], we analyzed whether the pathway was required for *P. acnes*‐induced mitochondrial biogenesis impairment. The expression of SIRT‐1 and the phosphorylation of AMPK were inhibited dramatically after *P. acnes* infection, and the mitochondrial biogenesis‐related factors, aggrecan, and collagen II declined subsequently (Fig. 8A,B). To verify the role of SIRT‐1, the specific SIRT‐1 activator SRT‐1720 (5 μm) was administered to activate cocultured NPCs, and we recognized that SIRT‐1 activity rose significantly (Fig. 8C) following the elevated expression of mitochondrial biogenesis‐related factors, despite the fact that SIRT‐1 and p‐AMPK expression was not affected (Fig. 8D,E). Moreover, treatment with SRT‐1720 enhanced the mtDNA/nDNA ratio (Fig. 8F) and the mean fluorescence intensity of MitoTracker Deep Red staining (Fig. 8G) as an index of improved mitochondrial biogenesis, but this improvement was attenuated by PGC‐1α and TFAM siRNA. The Seahorse mitochondrial stress analysis yielded similar results (Fig. 8H), with data collectively suggesting that inhibition of SIRT‐1 by *P. acnes* was the reason for mitochondrial biogenesis impairment.

Activation of SIRT‐1 also reversed the reduction in aggrecan and collagen II elicited by *P. acnes*. However, this effect was reversed by abrogation with PGC‐1α and TFAM (Fig. 8D,E), consistent with our immunofluorescence findings (Fig. 8I). Next, we further evaluated the *in vivo* efficacy of SIRT‐1 activation in mitigating IVDD caused by long‐term colonization with *P. acnes*. The activity detection of SIRT‐1 indicated that the SIRT‐1 activity in the nucleus pulposus tissue significantly increased after the injection of SRT‐1720 (Fig. 8J). MRI and CT analyses demonstrated that enhanced SIRT‐1 activity significantly attenuated disc degeneration and restored intervertebral disc height (Fig. 8K). Histological assessment using H&E and safranin O staining further confirmed that SIRT‐1 activation effectively preserved the structural integrity of the NP (Fig. 8L). In summary, our *in vitro* and *in vivo* findings demonstrate that *P. acnes* inhibits SIRT‐1, thereby inducing impairment of mitochondrial biogenesis and phagocytic activity in NPCs, which ultimately contributes to the development of infectious intervertebral disc degeneration.

**Fig. 9:**
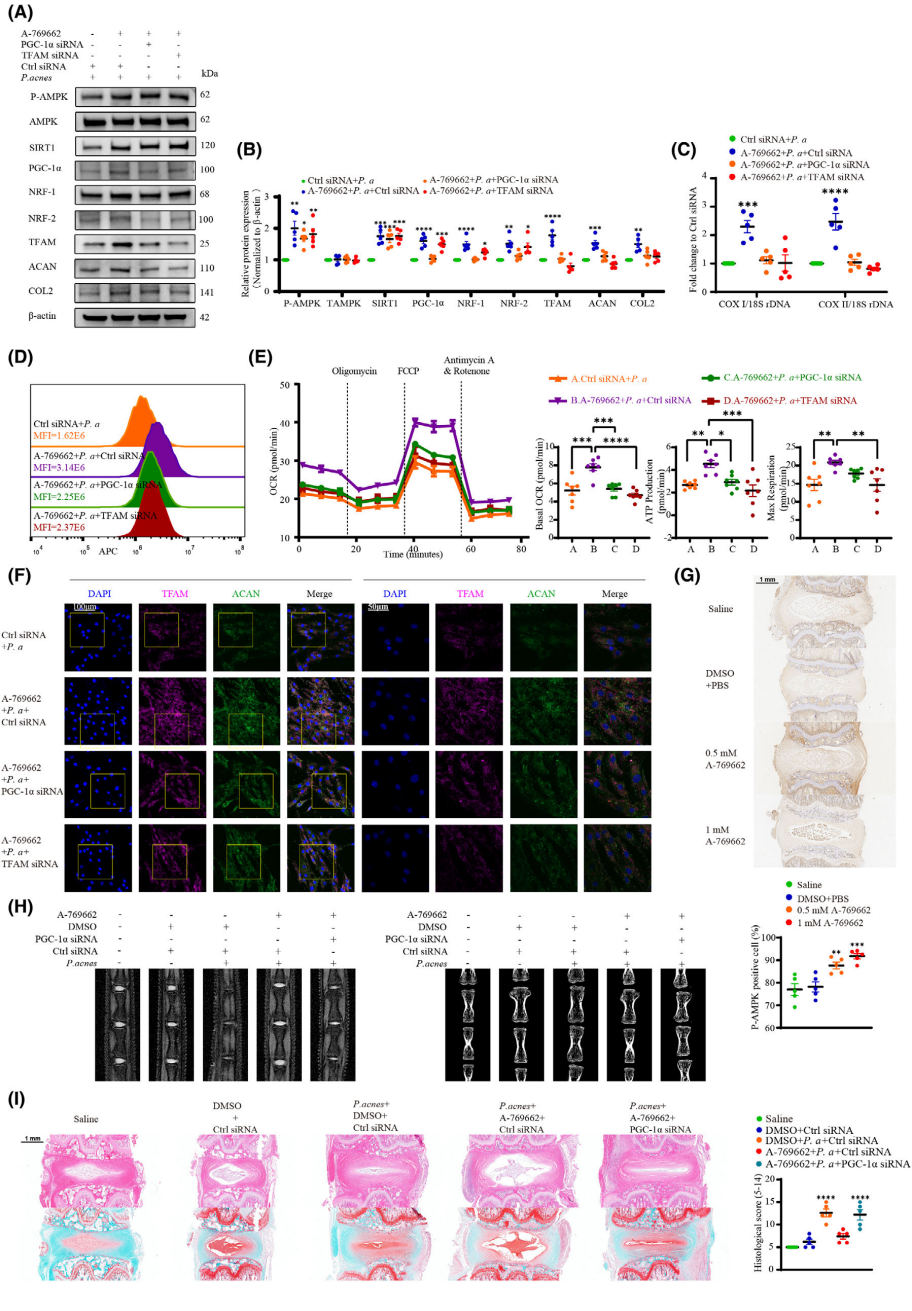
*P. acnes* impairs mitochondrial biogenesis of NPCs via the AMPK/SIRT‐1/PGC‐1α‐signaling pathway. (A and B) Western blot (A) and quantitative analyses (B) of p‐AMPK, total AMPK, SIRT‐1, mitochondrial biogenesis‐related factors, aggrecan, and collagen II. NPCs were pretreated with or without A‐769662 (0.1 mm) and siRNA, and then infected with *P. acnes* for 72 h. (C and D) Mitochondrial DNA content and (C) fluorescence histogram of mitochondrial mass (D) in NPCs after pretreatment with or without A‐769662 (0.1 mm) and siRNA before infection with *P. acnes* for 72 h. (E) OCR profiles, changes in basal OCR, ATP production, and maximal respiration of NPCs after pretreatment with or without A‐769662 (0.1 mm) and siRNA before incubation with *P. acnes* for 72 h (*N* = 7). (F) Immunofluorescence analysis of TFAM and aggrecan in NPCs. NPCs were pretreated with or without A‐769662 (0.1 mm) and siRNA, and then infected with *P. acnes* for 72 h; the right panels show magnified insets (Scale bar: 100/50 μm). (G) IHC staining for p‐AMPK and quantitative analysis in serial sections of rat caudal IVDs (Scale bar: 1 mm). (H) MRI analysis and Micro‐CT analysis of caudal discs. (I) HE staining, safranin O staining, and statistical graphs of histological scores in serial sections of rat caudal IVDs (Scale bar: 1 mm). *N* = 5, *P < 0.05, **P < 0.01, ***P < 0.001, ****P < 0.0001; values were analyzed using one‐way ANOVA; data are presented as the mean ± SEM. IVDs, intervertebral discs; NPCs, nucleus pulposus cells; OCR, oxygen consumption rate; *P. acnes/P. a*, *Propionibacterium acnes*.

To determine whether pharmacologic AMPK activation ameliorated the established impairments in mitochondrial biogenesis and phagocytic function, cocultured NPCs were treated with A‐769662 (0.1 mm), and the results depicted an increased phosphorylation of AMPKα and expression of SIRT‐1 and mitochondrial biogenesis‐related factors (Fig. 9A,B). However, in NPCs with PGC‐1α/TFAM knockdown, the overexpression of AMPK reflected a diminished capacity to upregulate the expression of mitochondrial biogenesis‐related factors. We also obtained similar results for both the mtDNA/nDNA ratio (Fig. 9C) and MitoTracker Deep Red staining (Fig. 9D). The results of the Seahorse mitochondrial stress test further confirmed that p‐AMPK improved basal OCR, ATP production, and max OCR of NPCs, and that these modulations were dependent on downstream PGC‐1α‐TFAM‐pathway components (Fig. 9E).

The overexpression of aggrecan and collagen II (Fig. 9A,B) proved that phosphorylation of AMPKα significantly ameliorated *P. acnes*‐induced infectious disc degeneration and that this process required the activation of p‐AMPK in mitochondrial biogenesis, and immunofluorescence analysis yielded similar findings (Fig. 9F). Subsequently, we sought to validate the role of AMPK in IVDD induced by long‐term colonization with *P. acnes in vivo*. IHC analysis demonstrated that intradiscal administration of A‐769662 significantly increased the number of p‐AMPK‐positive cells within the NP (Fig. 9G). MRI and CT assessments revealed that AMPK activation markedly attenuated both disc degeneration and the loss of intervertebral disc height caused by *P. acnes* (Fig. 9H). Histological evaluation using H&E and safranin O staining further confirmed that AMPK activation effectively mitigated structural damage to the NP (Fig. 9I). Collectively, these experimental findings indicate that *P. acnes* impairs mitochondrial biogenesis in NPCs both *in vitro* and *in vivo* via the AMPK/SIRT‐1/PGC‐1α signaling pathway.

## Discussion

We have verified the novel immune capabilities of NPCs in our previous study [20], and confirmed that NPCs have the ability to phagocytize bacteria and to exert microbicidal activity via the induction of phagolysosome formation. These data suggest that there exists a functionality of NPCs in defending against bacterial IVD infections. In the context of the ‘human microbial community,’ the bacterial etiology of IVDD has been proposed and has gained increasing attention [4, 16]. *P. acnes* has been found to colonize the IVD latently and to cause IVDD through a variety of mechanisms. Hence, the aforementioned two reports have highlighted an intriguing issue: why do NPCs only play a limited microbicidal role in the process of *P. acnes* infection? In this study, we demonstrated for the first time that *P. acnes* inhibited the bactericidal function of NPCs by impairing mitochondrial biogenesis and hindering immune processes that mitochondria involved, facilitating the harboring of *P. acnes* in IVDs for an extended period and inducing IVDD.

To reconfirm the fact that *P. acnes* resided in IVDs, we initially collected 87 patients' disc tissues and observed *P. acnes* colonization in the IVD, with a prevalence of 20.69%, similar to our previous report [28]. Based on our statistical results, we found an intriguing outcome depicting young IVDD patients as more susceptible to *P. acnes* infections than elderly patients, suggesting that *P. acnes* infection accelerated the progression of disc degeneration. Through our analysis of Pfirrmann grade, MRI index, and extracellular matrix protein expression, we further verified infection with *P. acnes* as a novel etiology in inducing IVDD.

IVDs are immunologically privileged structures with little or no direct vasculature, particularly the central nucleus pulposus (NP), where access to immune cells may be more limited than in most other tissues [23, 29]. Regarding microbicidal phagocytosis of NPCs, our previous work confirmed that NPCs function as non‐professional phagocytes against *Staphylococcus aureus* [20]. In the present study, by examining the phagocytosis of NPCs for *P. acnes* and latex beads, we confirmed that NPCs can phagocytose *P. acnes* and form phagocytic lysosomes to eliminate *P. acnes*. It was also demonstrated that the bactericidal function of NPC against *P. acnes* is contingent upon the mROS transported by mitochondrial‐derived vesicles (MDVs) to lysosomes. However, compared with non‐viable *P. acnes*, the active *P. acnes* significantly diminished the co‐localization ratio of mROS and lysosomes in NPC, impaired the bactericidal function of phagosomes, and markedly reduced the capacity of NPCs to engulf *P. acnes*. This suggested that the presence of viable *P. acnes* further attenuated the sustained bactericidal efficacy of lysosomes, potentially contributing to the persistent colonization of *P. acnes* in IVDs.

Therefore, to explore the mechanism underlying the impairment of NPC antimicrobial function, we conducted an RNA‐seq experiment of both *P. acnes*‐positive and *P. acnes*‐negative groups and uncovered a significant decrease in PGC‐1α expression in the *P. acnes*‐positive group. We know that PGC‐1α is a key factor in mitochondrial biogenesis that maintains cellular mitochondrial quantity and quality and ensures mitochondrial homeostasis [24]. A number of previous studies have indicated that mitochondrial dysfunction exerts a significant impact on the microbicidal function of macrophages [25, 26]; however, it remains uncertain whether *P. acnes* impairs phagocytosis of NPCs by damaging mitochondrial biogenesis. We herein observed that the attenuated expression of PGC‐1α and downstream factors (NRF1/2, TFAM), impaired mitochondrial quality, and reduced mitochondrial quantity in *P. acnes*‐positive samples. The Seahorse results also revealed that *P. acnes* disrupted the function of oxidative phosphorylation and altered energy metabolism. Furthermore, overexpression of PGC‐1α reversed the inhibitory effects of *P. acnes* on mitochondrial biogenesis and preserved the homeostasis of mitochondrial and energy metabolism. Thus, these results demonstrated that *P. acnes* hampered mitochondrial biogenesis and interfered with oxidative phosphorylation by suppressing PGC‐1α in NPCs.

We further explored the causal relationship between damaged mitochondrial biogenesis and impaired bactericidal function in NPCs infected with *P. acnes*. Mitochondrial homeostasis is the prerequisite for the proper physiological function of mitochondria. While *P. acnes* infection resulted in an overall increase in mROS within NPCs, it concurrently diminished the proportion of mROS that co‐localize with lysosomes. We hypothesized that the impairment of mitochondrial quality might impede the delivery of mROS to lysosomes via MDVs. Following the restoration of mitochondrial quality in NPCs impaired by *P. acnes* through overexpression of PGC‐1α, we observed a significant increase in the proportion of mROS co‐localizing with lysosomes, suggesting that *P. acnes* undermines lysosomal microbicidal function by inhibiting mitochondrial biogenesis.

While we recognize that oxidative phosphorylation in IVD cell biology remains an issue of debate [30], the general consensus is that NPCs require oxidative phosphorylation and ATP to remain healthy and active [31]. Additionally, the phagocytotic process requires the consumption of a large number of ATPs [32]. Hence, we proposed a reasonable hypothesis in which the decreased oxidative phosphorylation elicited by mitochondrial biogenesis damage in NPCs infected with *P. acnes* would be expected to lead to a significant diminution in ATP production, which in turn would lead to a reduction in cellular phagocytosis and bactericidal function. By regulating mitochondrial biogenesis and oxidative phosphorylation in NPCs, we demonstrated for the first time that mitochondrial biogenesis was essential for the maintenance of phagocytosis in NPCs, which was achieved by promoting oxidative phosphorylation. Mitochondrial biogenesis overexpression reversed the phagocytotic function inhibited by *P. acnes*, promoted the clearance of *P. acnes*, and prevented disc degeneration induced by long‐term *P. acnes* colonization. However, this effect was blocked by interference with mitochondrial biogenesis factors and oxidative phosphorylation. These results not only revealed the potential of mitochondrial biogenesis in modulating immune function but also shed new light on the unique significance of oxidative phosphorylation for NPCs. In addition, we uncovered a crucial mechanism by which *P. acnes* evades phagocytosis, providing new strategies for the prevention and treatment of other *P. acnes*‐induced diseases.

We subsequently explored the mechanisms underlying this novel pathological process. We recognize that AMPK is a major regulator of mitochondrial biogenesis and regulates intracellular energy metabolism [33], and that the silent information regulator protein (Sir2) homolog SIRT‐1 constitutes another activator of PGC‐1α via deacetylation [34]. NPCs are a type of chondrocyte‐like cell, and numerous mechanisms reflect similarities to articular chondrocytes [35]; for example, in chondrocytes, the AMPK/SIRT‐1/PGC‐1α pathway has been verified [36]. In this study, we observed reduced AMPK phosphorylation and SIRT‐1 expression in *P. acnes*‐infected NPCs, and pharmacological activation of AMPK and SIRT‐1 significantly reversed the reduction in mitochondrial biogenesis‐related factors, improved oxidative metabolism, and ultimately reversed *P. acnes*‐induced disc degeneration. In summary, *P. acnes* impaired the mitochondrial biogenesis of NPCs via the AMPK/SIRT‐1/PGC‐1α signaling pathway (Graphical abstract).

In summary, in this study, we demonstrated that NPCs acted as non‐professional phagocytic cells to eliminate latent infected *P. acnes*. Mitochondrial biogenesis augmented the bactericidal activity of lysosomes by promoting mROS transportation through the regulation of mitochondrial quality and enhanced the phagocytic capacity of NPCs by enhancing oxidative phosphorylation function. *P. acnes* inhibits the mitochondrial biogenesis of NPCs through the AMPK/SIRT‐1/PGC‐1α pathway, impairs the bactericidal function of NPCs, and then colonizes the intervertebral disc for a prolonged period, ultimately leading to IVDD. These results provide novel insights into the immune function of intervertebral discs, elucidate the mechanism by which *P. acnes* evades the phagocyte, and identify new targets for the treatment of disc infections.

## Materials and methods

### Reagents

The selective AMPK pharmacologic activator A‐769662 and the selective SIRT‐1 activator SRT‐1720 were obtained from Selleck (Houston, TX, USA). The NecroX‐5 was obtained from MCE (Shanghai, Shanghai, China). In brief, NPCs were treated with 5 μm SRT‐1720 or 0.1 mm A‐769662 or 10 μm NecroX‐5 12 h before coculture with *P. acnes*. For *in vivo* experiments, SRT‐1720 and A‐769662 were initially dissolved in DMSO and subsequently diluted with saline to obtain final concentrations of 25 to 50 μm for SRT‐1720 and 0.5 to 1 mm for A‐769662. Then, a volume of 1 μL of the solution was injected into the rat intervertebral discs.

### Patients and tissue harvesting

The study was carried out in accordance with the guidelines of the Declaration of Helsinki. Experiments using human materials were approved by the Ruijin Hospital Ethics Committee of Shanghai Jiaotong University School of Medicine (2013‐Ethics Committee‐No.60). All participating patients have duly signed written informed consent forms.

Patient samples were obtained at Ruijin Hospital between July 2020 and July 2022, and the study involved 87 patients aged 17–61 years. The inclusion criteria were as follows: (a) possession of single‐level lumbar disc herniation at L4/L5 as confirmed by MRI and CT; (b) radiological examination matching clinical symptoms of low back pain and sciatica; and (c) conservative treatment shown to be ineffective for at least 3 months. The exclusion criteria were as follows: spinal fractures, spinal cord injuries, trauma, spinal tumors, spinal infections, and lumbar kyphosis (data on patient characteristics are depicted in Table 1). MR images and disc tissues were collected after discectomy for the subsequent experiments.

### Cocultures of NPCs and *P. acnes in vitro*

Per our previous protocol [20], we cultured primary NPCs obtained from clinical disc tissues in six‐well plates. *P. acnes* was then added to the cell culture at a 100 : 1 MOI without antibiotics. After 36 to 72 h, cocultured cells were washed three times with PBS and prepared for further experiments.

### Inoculation of *P. acnes* into caudal rat IVDs *in vivo*

Male Sprague–Dawley rats at 8 weeks of age were obtained from Charles River Laboratories. All rats were housed in a controlled environment maintained at 23–26 °C, with 60–80% humidity and a 12‐h light/dark cycle. Tap water was available to the rats at all times during the experiments.

Based on our previous protocol [16], a micro‐syringe with a 28‐gauge needle attached (Hamilton, Reno, NV, USA) was used to inoculate *P. acnes* (2 μL, OD600 = 3.0) into the NP. All animal experiments were performed following the protocol approved by the Shanghai Jiao Tong University (SJTU) Animal Care and Use Committee (IACUC protocol number SYXK [Shanghai] 2011‐0113) in accordance with the Ministry of Science and Technology of the People's Republic of China Animal Care guidelines. Rats were anesthetized by intraperitoneal injection of ketamine (70 mg·kg^−1^) and diazepam (7 mg·kg^−1^) and euthanized by intraperitoneal injection of three times the above doses of ketamine and diazepam. All surgeries were conducted under anesthesia, and all efforts were taken to reduce suffering.

### Western blot analysis

To conduct western blot analysis, total sample proteins were obtained in ice‐cold RIPA, separated by SDS/PAGE, and subsequently transferred to nylon membranes. The bands were then incubated separately with the following primary antibodies: collagen II (dilution, 1 : 800; cat. no. ab34712, Abcam, Cambridge, CA, USA), aggrecan (1 : 800; cat. no. ab36861, Abcam), PGC‐1α (1 : 800; cat. no. ab191838, Abcam), NRF1 (1 : 1000; cat. no. 46743, CST, Danvers, MA, USA), NRF2 (1 : 800; cat. no. 33649, CST), TFAM (1 : 1000; cat. no. ab272885, Abcam), p‐AMPK (1 : 1000; cat. no. 50081, CST), AMPK (1 : 1000; cat. no. 5831, CST), SIRT‐1 (1 : 1000; cat. no. 8469, CST), and β‐actin (1 : 2000; cat. no. ab6276, Abcam). The membranes were subsequently probed separately with the following secondary antibodies: goat anti‐rabbit IgG (1 : 2000; cat. no. 7074, CST) and horse anti‐mouse IgG (1 : 2000; cat. no. 7076, CST) at room temperature for 1 h. Chemiluminescence (Pierce Biotechnology, Rockford, IL, USA) was used to visualize the band, and Fusion FX7 (Vilber Lourmat, Collegien, Seine‐et‐Marne, France) was adopted to analyze the images.

### Bacterial killing assay

NPCs were plated at 1 × 10^6^ cells per well in six‐well plates, and *P. acnes* were added at an MOI of 50 in NPCs or blank groups that contained only culture medium; we then cocultured for 0, 12, 24, 36, 48, 60, and 72 h. The culture medium was then aspirated, and the six‐well plates were rinsed five times with ice‐cold PBS to remove the remaining *P. acnes*. All of the culture medium and PBS rinses were collected from each well, centrifuged at 6000 ***g*** for 5 min, and resuspended with the same amount of PBS as the medium. We determined viable cell counts with the gradient dilution method, with the different dilutions (1 : 10^2^, 1 : 10^3^, 1 : 10^4^, and 1 : 10^5^) added to the agar LB medium. *P. acnes* was quantified by counting the number of colony‐forming units (CFU) at each time point and calculated using log (#CFU at time X).

### Measurement of lysosomes and mROS

To assess the co‐localization of ingested *P. acnes* with lysosomes, we stained *P. acnes* using BacLight (cat. no. B35000, Thermo Fisher Scientific, Waltham, MA, USA) and cocultured it with NPCs for varying durations. Subsequently, we removed the culture medium, washed the cells five times with ice‐cold PBS, and treated them with gentamicin to eliminate extracellular *P. acnes*. The cells were then stained with a LysoTracker (cat. no. L7528, Thermo Fisher Scientific) and imaged using a laser confocal microscope.

To evaluate the co‐localization of mROS with lysosomes, NPCs were stained with LysoTracker (cat. no. L7526, Thermo Fisher Scientific) and MitoSOX (cat. no. M36009, Thermo Fisher Scientific) following their coculture with *P. acnes*. Imaging was performed using a laser confocal microscope. To quantify total mROS levels in the cells, trypsin was employed to digest NPCs post‐MitoSOX staining, followed by resuspension in DMEM/F12 for analysis via flow cytometry.

### Phagocytotic function assay

To detect phagocytotic function, NPCs and *P. acnes* were cocultured for 36 to 72 h, and inactivated *P. acnes* was added to NPCs as a control. After removing the culture medium, gentamycin was used to eliminate extracellular bacteria, and NPCs were rinsed five times with ice‐cold PBS. The NPCs were then incubated with DMEM/F12 containing 2‰ latex beads (no. L4530, Sigma Aldrich, St. Louis, MO, USA) for 8 h and rinsed five times with ice‐cold PBS to remove excess beads. The internalized fluorescent green latex beads were measured with a Beckman CytoFLEX flow cytometer (Beckman, Brea, CA, USA) or a Zeiss LSM800 (Carl Zeiss, Oberkochen, Baden‐Württemberg, Germany). For laser confocal detection, DAPI (cat. no. G1012, Servicebio, Wuhan, China) and Actin‐Tracker (cat. no. C2203S, Beyotime, Beijing, China) were used to stain nuclei and cytoskeletons, respectively.

### Electron microscopy

NPCs previously cocultured with *P. acnes* underwent fixation with 2.5% glutaraldehyde in 0.1 m phosphate buffer (pH 7.4) for 4 h, and conventional electron microscopy was conducted. Ultrathin sections were stained with uranyl acetate and lead citrate, and photographed by an H7700 electron microscope (Hitachi, Tokyo, Japan).

### Real‐time PCR for mRNA expression

We utilized TRIzol (Invitrogen, Carlsbad, CA, USA) to extract total RNA from the tissue or NP cells, and a reverse‐transcription reagent kit (cat. no. DRR047A, TaKaRa, Kusatsu, Shiga Prefecture, Japan) was applied to synthesize cDNA. Real‐time PCR was performed with a PCR detection kit (cat. no. DRR081A, TaKaRa) and ABI 7500 Sequence Detection System to analyze the expression of genes. The housekeeping gene β‐actin served as a control, and each reaction was performed in triplicate. We calculated expression using the $2^{-\Delta \Delta C_{t}}$ method. The primers (Sangon Biotech, Shanghai, China) we applied for mRNA expression analysis are listed in Table 2.

**Table 2:** List of primers for real‐time PCR.

Gene	Organism	Forward primer (5′–3′)	Reverse primer (5′–3′)
PGC‐1α	Human	TCCAGGTCAAGATCAAGGTCTCCAG	GTGCGGTGTCTGTAGTGGCTTG
NRF1	Human	AATTATTCTGCCGTGGCTGATGGAG	CCTCTGATGCTTGCGTCGTCTG
NRF2	Human	AGTCCAGAAGCCAAACTGACAGAAG	GGAGAGGATGCTGCTGAAGGAATC
TFAM	Human	TGGCGTTTCTCCGAAGCATGTG	TGCCAAGACAGATGAAAACCACCTC
β‐actin	Human	GTCATTCCAAATATGAGATGCGT	GCTATCACCTCCCCTGTGTG
COX1	Human	GGCCTGACTGGCATTGTATT	TGGCGTAGGTTTGGTCTAGG
COX2	Human	CCCCACATTAGGCTTAAAAACAGAT	TATACCCCCGGTCGTGTAGC
18S rDNA	Human	TAGAGGGACAAGTGGCGTTC	CGCTGAGCCAGTCAGTGT
COX1	Rat	AGCCGGGGTGTCTTCTATCT	CTTCTGGGTGGCCGAAGAAT
COX2	Rat	CACAAGCACAATAGACGCCC	TGTAGCTTGGTTTAGGCGGC
β‐actin	Rat	CTGCTCTTTCCCAGATGAGG	CCACAGCACTGTAGGGGTTT

### Real‐time PCR for mitochondrial DNA content

Total DNA was isolated from the tissue or NP cells using the QIAamp DNA Mini Kit (cat. no. 51306, Qiagen, Hilden, North Rhine‐Westphalia, Germany) following the manufacturer's instructions. The ratio of COX1 and COX2 DNA copies to 18S rDNA or β‐actin represented the relative mitochondrial copy number. Expression was analyzed with the $2^{-\Delta \Delta C_{t}}$ method. The primers (Sangon Biotech) for mitochondrial DNA content analysis are listed in Table 2.

### Flow‐cytometric analysis of mitochondrial mass

For mitochondrial mass analysis, NPCs were incubated with 200 nm MitoTracker Deep Red (cat. no. M22426, Thermo Fisher Scientific) for 20 min after *P. acnes* treatment and PBS rinses. The cells were digested with trypsin, centrifuged at 300 ***g***, and resuspended in 1% PBS. We used the Beckman CytoFLEX flow cytometer to conduct flow‐cytometric analysis and processed the data with flowjo 10 software (TreeStar, Ashland, OR, USA), with 10 000 events collected for each sample.

### Lactate assay

The concentration of lactate was assessed using the lactate colorimetric/fluorometric assay kit (cat. no. ab65330, Abcam), with the assay conducted following the manufacturer's instructions. The standard curve was generated with assay buffers supplied in the kit.

### Determination of ATP content

ATP content of NPCs and harvested tissue was measured using an ATP bioluminescence assay kit (cat. no. S0026, Beyotime), and the concentration of total protein was determined with a BCA Protein Assay Kit (cat. no. P0010S, Beyotime).

### Seahorse assay for metabolic determination

Congruent with previous research [37], NPCs were plated in Seahorse XF96 cell culture microplates (Agilent Technologies, Santa Clara, CA, USA) and then cocultured with *P. acnes* (MOI = 100). To analyze the OCR, cells were incubated with a mixture containing XF medium, glucose (10 mm), pyruvate (1 mm), and l‐glutamine (1 mm) for 1 h. The OCR was determined using the Seahorse XFe96 extracellular flux assay kit with XF calibrant. The cells were then supplemented with oligomycin (2.5 μm), FCCP (1 μm), and rotenone/antimycin A (0.5 μm) sequentially, and the measurement of OCR was performed using the Seahorse XFe96 analyzer (Agilent Technologies).

### Histology and immunohistochemistry (IHC)

We executed conventional methods to process specimens for IHC [38]. The caudal IVDs of the rat were fixed in 4% paraformaldehyde for 2 days and decalcified by 10% EDTA for 1 month. The caudal IVDs underwent routine paraffin embedding, sectioning, and deparaffinization sequentially. The sections were stained using a standard HE and safranin O‐fast green staining protocol. Two independent histopathology experts conducted blinded microscopic evaluations of nucleus pulposus, annulus fibrosus, and endplate tissues to assess cellular density and structural morphology. For IHC, the sections were incubated with TFAM (at a dilution of 1 : 200; cat. no. ab272885, Abcam) or p‐AMPK (1 : 1000; cat. no. 50081, CST) at 4 °C overnight. Nuclei were stained by hemalum (cat. No G1004, Servicebio), and the samples were ultimately viewed and captured under light microscopy (Carl Zeiss).

### Immunofluorescence

For *in vitro* work, *P. acnes*‐infected cells were fixed for 20 min with 4% paraformaldehyde and rinsed with PBS. The NPCs were permeabilized in 0.1% Triton X‐100/PBS (cat. no. T8787, Millipore Sigma, Burlington, MA, USA) for 10 min and blocked using 10% normal goat serum in PBS (cat. no. G9023, Millipore Sigma) for 30 min. The cells were thereafter incubated for 12 h at 4 °C with TFAM antibody (dilution of 1 : 500; cat. no. ab119684, Abcam) and aggrecan antibody (1 : 500; cat. no. 13880‐1‐AP, Proteintech, Wuhan, China). After three washes in PBS for 5 min each, cells were incubated with secondary antibody, goat anti‐rabbit‐Alexa Fluor 488 (1 : 400; cat. no. A‐11008, Thermo Fisher Scientific), goat anti‐mouse‐Alexa Fluor 555 (1 : 400; cat. no. A‐21422, Thermo Fisher Scientific), and goat anti‐mouse‐Alexa Fluor 647 (dilution of 1 : 400; cat. no. A‐21235, Thermo Fisher Scientific) for 1 h at room temperature. Cells were then treated with 2 μg·mL^−1^ DAPI (cat. no. G1012, Servicebio) for 5 min and washed in PBS. All images were observed using a Zeiss LSM800 (Carl Zeiss) equipped with ×20/×40 lenses and operated with zen blue software (Carl Zeiss).

*In vivo*, rat caudal disc sections were fixed in 4% paraformaldehyde for 2 days; decalcified by 10% EDTA for 4 weeks; underwent antigen retrieval; and were permeabilized, blocked, and sectioned at 5 μm. Immunofluorescence was performed with primary antibodies against collagen II (1 : 200; cat. no. ab34712, Abcam) and *P. acnes* (1 : 200; cat. no. D371‐3, MBL, Nagoya, Japan). After incubation with appropriate secondary antibodies at RT for 2 h and staining with DAPI (C1006, Beyotime) for 10 min, the sections were analyzed under a fluorescence microscope (Carl Zeiss).

### Small interfering RNA (siRNA) sequences

The siRNAs against PGC‐1α/TFAM mRNA were designed by GenePharma (Shanghai, China). The siRNA sequences were as follows: Human PGC‐1α, sense 5′‐GGU GCA GUG ACC AAU CAG ATT‐3′, anti‐sense 5′‐UCU GAU UGG UCA CUG CAC CTT‐3′; Human TFAM, sense 5′‐GUG GCA GGU AUA UAA AGA ATT‐3′, anti‐sense 5′‐UUC UUU AUA UAC CUG CCA CTT‐3′; and rat PGC‐1α, sense 5′‐GGC AGU AGA UCC UCU UCA ATT‐3′, anti‐sense 5′‐UUG AAG AGG AUC UAC UGC CTT‐3′. *In vitro*, NPCs were transfected with siRNA using Lipofectamine™ 3000 (Invitrogen). *In vivo*, PGC‐1α or control siRNA with Invivofectamine™ 3.0 (Invitrogen) was pre‐injected into the rat caudal discs 3 days before *P. acnes* inoculation. The efficiency of gene knockdown was assessed using a western blot 48 h after transfection.

### Lentiviral vector and adeno‐associated virus

We purchased the lentiviral vector (LV)‐PGC‐1α from GenePharma and puromycin from Sigma and used them to select stably transfected cells. The efficiency of gene overexpression was detected by a western blot 5 days after lentiviral vector transfection.

The adeno‐associated virus serotype 5 (AAV)‐PGC‐1α was designed by GenePharma. Two weeks before the inoculation of *P. acnes* into rat caudal discs, the AAV5 (1 μL, 2 × 10^13^ VG·mL^−1^) was directly injected into the NP of the tail discs with a 28‐gauge needle (Hamilton).

### Micro‐CT and MRI analysis

Five days after surgery, micro‐CT and MRI analyses were conducted on all rats after sacrifice. Micro‐CT was implemented by the high‐resolution micro‐CT scanner (Bruker, Billerica, MA, USA) for quantitative disc height evaluation, and prone‐position MRI was executed using a 3.0T system (GE) to obtain T2‐weighted images (repetition time, 1800 ms; echo time, 70 ms; field of view, 75 × 75 mm; and slice thickness, 2.0 mm).

### SIRT‐1 activity assay

We extracted nuclei with a Nucleus Extraction Kit (cat. no. C5034, Bioss, Beijing, China). SIRT‐1 activity was assessed using the SIRT‐1 Activity Assay Kit (ab156065, Abcam).

### Statistical analysis

Statistical analysis was performed using graphpad prism9 software (GraphPad Software, La Jolla, CA, USA). We collected data from three or more independent experiments and expressed them as the mean ± standard error of the mean or standard deviation. A two‐sided Student's *t* test was used to analyze the differences between the two groups, and one‐way analysis of variance (ANOVA) was performed to show differences among multiple groups. *P* < 0.05 was considered significantly different.

## Conflict of interest

The authors declare no conflict of interest.

## Author contributions

LR: writing – original draft, investigation, methodology. CL: writing – review, funding acquisition. JS: methodology, data curation. YZ: software. YJ: methodology. JZ: visualization. FZ: conceptualization. YL: writing – review, funding acquisition. WW: supervision, resources. PC: methodology, funding acquisition.

## Data Availability

The data of this study are available upon reasonable request.
